# ΔNp63 mediates cellular survival and metastasis in canine osteosarcoma

**DOI:** 10.18632/oncotarget.10406

**Published:** 2016-07-06

**Authors:** Maren Cam, Heather L. Gardner, Ryan D. Roberts, Joelle M. Fenger, Denis C. Guttridge, Cheryl A. London, Hakan Cam

**Affiliations:** ^1^ Center for Childhood Cancer and Blood Diseases, Nationwide Children's Hospital, Columbus, Ohio 43205, USA; ^2^ Department of Pediatrics, The Ohio State University College of Medicine, The Ohio State University, Columbus, Ohio 43210, USA; ^3^ Department of Veterinary Clinical Sciences and Biosciences, The Ohio State University, Columbus, Ohio 43210, USA; ^4^ Department of Molecular Virology, Immunology, and Medical Genetics, Human Cancer Genetics Program, The Ohio State University, Columbus, Ohio 43210, USA

**Keywords:** osteosarcoma, metastasis, p53 family member, deltaNp63, canine

## Abstract

*p63* is a structural homolog within the 53 family encoding two isoforms, ΔNp63 and TAp63. The oncogenic activity of ΔNp63 has been demonstrated in multiple cancers, however the underlying mechanisms that contribute to tumorigenesis are poorly characterized. Osteosarcoma (OSA) is the most common primary bone tumor in dogs, exhibiting clinical behavior and molecular biology essentially identical to its human counterpart. The purpose of this study was to evaluate the potential contribution of ΔNp63 to the biology of canine OSA. As demonstrated by qRT-PCR, nearly all canine OSA cell lines and tissues overexpressed ΔNp63 relative to normal control osteoblasts. Inhibition of ΔNp63 by RNAi selectively induced apoptosis in the OSA cell lines overexpressing ΔNp63. Knockdown of ΔNp63 upregulated expression of the proapoptotic Bcl-2 family members Puma and Noxa independent of p53. However the effects of ΔNp63 required transactivating isoforms of p73, suggesting that ΔNp63 promotes survival in OSA by repressing p73-dependent apoptosis. In addition, ΔNp63 modulated angiogenesis and invasion through its effects on VEGF-A and IL-8 expression, and STAT3 phosphorylation. Lastly, the capacity of canine OSA cell lines to form pulmonary metastasis was directly related to expression levels of ΔNp63 in a murine model of metastatic OSA. Together, these data demonstrate that ΔNp63 inhibits apoptosis and promotes metastasis, supporting continued evaluation of this oncogene as a therapeutic target in both human and canine OSA.

## INTRODUCTION

Osteosarcoma (OSA) is the most common malignant primary bone tumor in humans and dogs. It occurs predominantly in growing adolescents and young adults, with a peak incidence at 15–19 years of age [1]. Advances in neo-adjuvant chemotherapy over the past 30 years have increased the 5-year overall survival rate of patients from 20% to 70% [2, 3]. However, patients with metastatic disease at diagnosis have a guarded prognosis, with 5-year overall survival rate of 10-30% [3–5]. In contrast to human OSA, canine OSA is more common in older dogs. Similar to its human counterpart, adjuvant chemotherapy has improved median survival times from 3-4 to 10-12 months, however 90% of dogs are euthanized due to progressive metastatic disease within 2 years of diagnosis [6, 7]. Importantly, canine OSA exhibits essentially identical clinical behavior to its human counterpart, and demonstrates molecular aberrations indistinguishable supporting the notion that canine OSA is a relevant model to human osteosarcoma that may serve as relevant targets for therapeutic intervention [8, 9]. Taken together, as metastatic disease is the primary cause of death of patients affected by OSA, characterization of the pathways that contribute to this process is necessary for the successful incorporation of novel therapeutics into treatment regimens.

Both p63 and p73 are transcription factors belonging to the p53 family. Several isoforms of p63 and p73 exist with high sequence and structural homology to those found with p53 [10–12]. The p63 gene generates transcripts encoding two major classes of protein isoforms through the use of alternative promoters: TAp63 and ΔNp63. TAp63 contains an full-length N-terminal transactivation (TA) domain, whereas ΔNp63 contains a truncated TA domain [11]. Recent evidence suggests that these alternative promoters endow p63 isoforms with differential functions. TAp63^−/−^ mice develop highly metastatic tumors, including mammary and pulmonary carcinomas, as well as squamous cell carcinoma [13]. TAp63 isoforms suppress metastasis through induction of senescence [14] and transcriptional activation of *Dicer1* and *mir-130b*, providing support for the metastatic phenotype associated with cells lacking *TAp63*[13]. Moreover, under conditions of DNA damage, coordinated activity of the tumor suppressors TAp53 and TAp63 is necessary to inhibit mTORC1 signaling, thus suppressing cell cycle progression and translation [15]. Taken together, several studies indicate that the TA isoforms act as tumor suppressors capable of efficiently transactivating different p53 responsive genes, largely mimicking p53-suppressive activities [16].

ΔN isoforms, including ΔNp63, oppose TAp53-, TAp63-, and p73-mediated transcription, and therefore apoptosis and cell cycle arrest, by blocking target promoters or by forming inactive heteromeric complexes [17]. In this regard, aberrant expression of N-terminally truncated isoforms of all three family members has gained attention, resulting in emergence of their crucial antagonistic role against the tumor suppressor activity of full-length p53 family members. As such, ΔN variants act as survival factors that confer drug resistance to tumor cells expressing wild-type p53 or p63/p73 by counteracting the growth-suppressive and cell death–inducing properties of p53 and p63/p73 [11, 18–20]. Importantly, the ratio between ΔN variants and full-length p53 family members influences tumor cell survival by modulating the functional growth inhibitory roles of p53 and p63/p73. Recent evidence suggests that the oncogenic function of N-truncated variants is more complex by eliciting a number of behaviors independent of any dominant-negative activity [17, 20, 21]. For example, the chromatin remodeler Lsh, essential for stem-like proliferation and tumorigenesis, has been identified as a target of ΔNp63α [22]. Additionally, ΔNp63 has been shown to promote pediatric neuroblastoma and OSA by regulating tumor angiogenesis independent of any dominant negative inhibitory activities [21].

While the p63 gene is rarely mutated in human tumors, the ΔNp63 isoform is often aberrantly expressed in several cancers and its high expression associated with a poor prognosis [16, 23–25]. The potential contribution of ΔNp63 to the biology of human OSA has not been well investigated. Given that canine OSA recapitulates the heterogeneity and biology of human OSA, preclinical interrogation of novel molecular drivers of disease can be performed in the setting of the canine disease. As such, the purpose of this study was to evaluate the expression and function of ΔNp63 in canine OSA. Our data demonstrate that ΔNp63 is frequently overexpressed in canine OSA cell lines and tumor samples. Importantly, we find that ΔNp63 promotes tumor cell survival through suppression of a p73-dependent proapoptotic transcriptional program. Furthermore, using gene knockdown and overexpression approaches, we demonstrate that ΔNp63 regulates cellular invasion, migration and angiogenesis by activating STAT3 in canine OSA cells. Lastly, correlation of ΔNp63 expression with increasing pulmonary metastatic foci in a murine model supports the notion that ΔNp63 is important for the metastatic phenotype in OSA.

## RESULTS

### Canine osteosarcoma expresses high levels of ΔNp63

**Figure 1:**
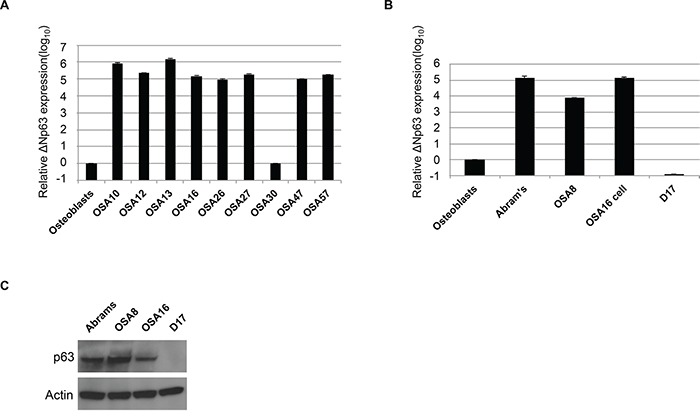
Canine osteosarcoma overexpresses ΔNp63 **A.** qRT-PCR was used to assay ΔNp63 mRNA levels in primary canine OSA tumors relative to control osteoblasts. Total RNA was reverse-transcribed and subjected to real-time PCR with probes specific for ΔNp63. Results were normalized to GAPDH. **B.** qRT-PCR was used to assay ΔNp63 mRNA levels in canine osteosarcoma cell lines relative to control osteoblasts. Results were normalized to GAPDH. **C.** Immunoblot analysis of p63 protein expression in osteosarcoma cell lines. Cell extracts were analyzed by western blot with antibodies as shown.

To determine ΔNp63 expression level in canine OSA, we analyzed nine primary canine OSA tumors and four different OSA cell lines. As shown in Figure 1A, qRT-PCR assays designed to distinguish TA from ΔN isoforms of p63 demonstrated that approximately 90% of primary OSA tumors express high levels of the ΔNp63 isoform when compared to that found in normal osteoblasts. Furthermore, ΔNp63 was highly expressed in three out of the four cell lines examined (Figure 1B), ranging from 3 to 5 logs higher expression relative to normal osteoblasts. Protein expression of ΔNp63 in canine OSA cell lines correlated well with expression of the ΔNp63 mRNA transcript (Figure 1C). These data show that ΔNp63 is highly overexpressed in both primary canine OSA tumors and OSA cell lines.

### Inhibition of ΔNp63 in canine osteosarcoma induces apoptosis

**Figure 2:**
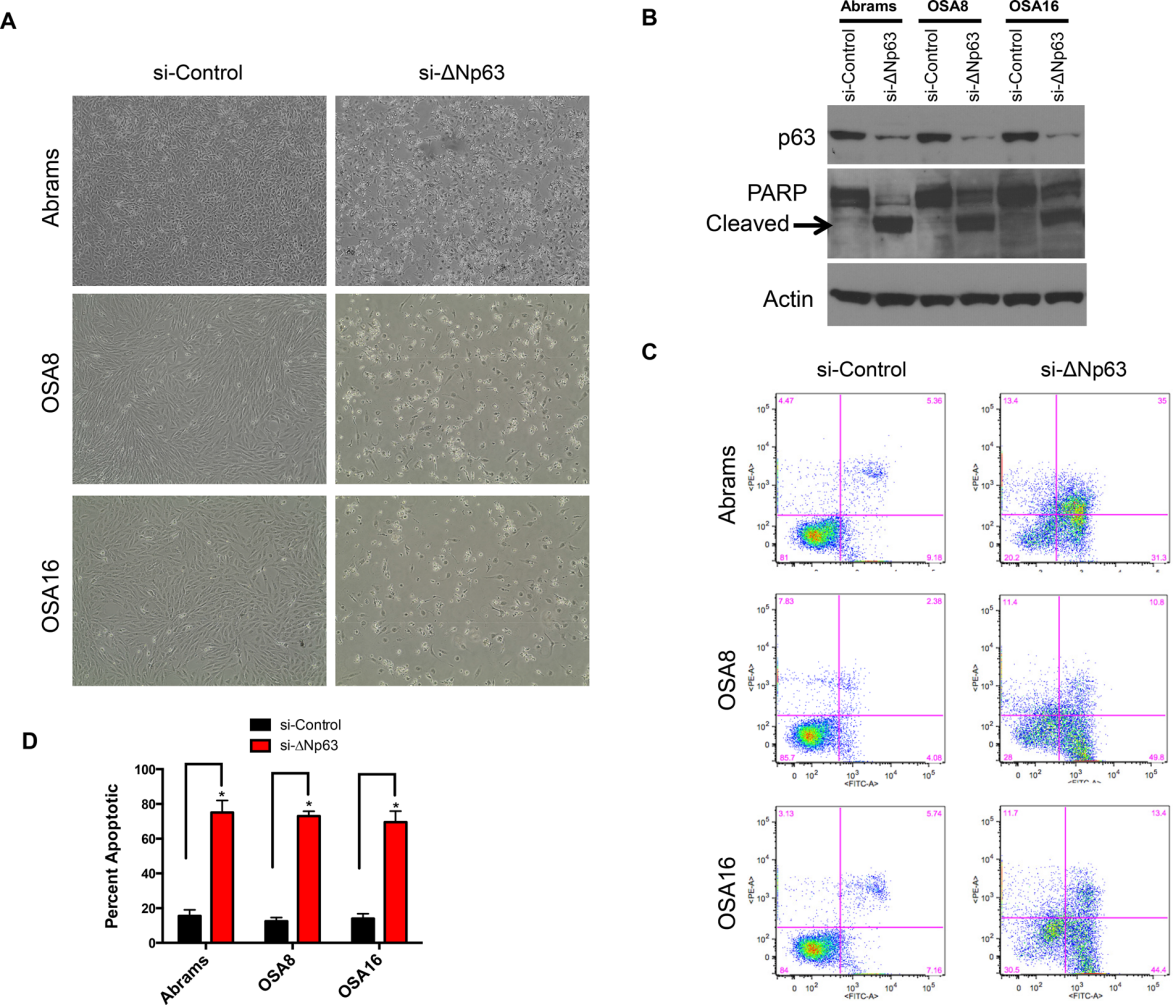
Knockdown of endogenous ΔNp63 induces apoptosis **A.** Loss of canine cells following infection with the si-ΔNp63 compared with the control siRNA. Representative fields were photographed 72 hr following siRNA transfection. **B.** Knockdown of ΔNp63 induces PARP cleavage in canine osteosarcoma cells. Cell extracts were harvested from indicated cell lines 72 hr following infection with si-ΔNp63 or control siRNA and analyzed by western blot with antibodies as shown. **C.** Induction of apoptosis in canine osteosarcoma cells following transfection with either si-ΔNp63 or control siRNA. Unfixed cells were stained with annexin V and propidium iodide (PI) 72 hr following infection with the indicated siRNAs, then analyzed with flow cytometry. Numbers refer to the percent annexin V- and/or PI-positive cells (UL + UR + LR quadrants) in this representative experiment. **D.** Quantitation of annexin V- and/or PI-positive cells treated and analyzed as in C.

To investigate the cellular behaviors associated with ΔNp63 expression, endogenous ΔNp63 protein was downregulated using siRNA interference. In contrast to human OSA cells, in which inhibition of ΔNp63 significantly reduced cell proliferation and colony formation [21], loss of ΔNp63 in canine OSA cell lines induced cell death (Figure 2A) through apoptosis, as evidenced by cleaved PARP (Figure 2B). This finding was validated by using alternate siRNA targeting ΔNp63 (Supplementary Figure S1A). No significant effects on cell viability were observed at early time points (24–48hr) in Abrams or OSA8 cells following transfection with a ΔNp63 specific siRNA. Though, cleaved PARP was present in both Abrams and OSA8 cells at 72hr post-transfection (Supplementary Figure S1B).

To confirm the fraction of apoptotic cells, unfixed cells were stained with annexin V/propidium iodide (PI) for flow cytometric cell cycle analysis 72 hr following siRNA transfection. Figure 2C shows representative annexin V/PI profiles, and the data are summarized for all cell lines in Figure 2D. Approximately two-thirds of Abrams, OSA8 and OSA16 cells underwent apoptosis within 72 hr of transfection with the ΔNp63 specific siRNA (Figure 2C and 2D). As previously noted, no increase in cell death was observed following transfection of the control siRNA. Furthermore, no death was observed in the D17 cell line, which does not express ΔNp63, following transfection with any of the siRNAs directed against ΔNp63 (Supplementary Figure S2). These data suggest that specific downregulation of ΔNp63 triggers apoptotic cell death in canine OSA cell lines.

### ΔNp63 mediates apoptosis in a p73 dependent manner

**Figure 3:**
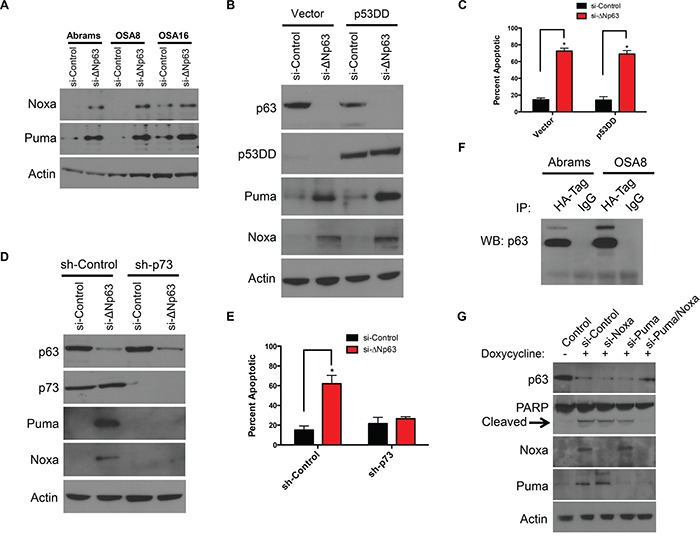
Puma and Noxa induction and cell death following ΔNp63 inhibition are p53 independent but require p73 **A.** Puma and Noxa protein expression correlates with ΔNp63 inhibition. Cell extracts were harvested from cell lines 72 hr following infection with a si-ΔNp63 or the control siRNA and analyzed by western blot with antibodies as shown. **B.** Puma and Noxa induction following ΔNp63 inhibition are not inhibited by p53DD expression. After Abrams cells were infected with the control vector or p53DD expressing lentiviral vectors, infected cells were selected with hygromycin. Abrams expressing either p53DD or the control lentiviral vector were treated with a si-ΔNp63 or the control siRNA for 72 hr. Subsequently, cell extracts were analyzed by western blot with antibodies as shown. **C.** Cell death following si-ΔNp63 is p53 independent. Quantitation of annexin V- and/or PI-positive cells treated and analyzed as in B. Error bars show standard deviation for two independent experiments. **D.** Puma and Noxa induction following inhibition of ΔNp63 require endogenous p73. Abrams cells were infected with the control shRNA or p73-directed shRNA expressing lentiviral vectors. Stable pools of Abrams infected with expressing a p73-directed shRNA or control shRNA were transfected with si-ΔNp63 or the control siRNA for 72 hr. Subsequently, cell extracts were analyzed by western blot with antibodies as shown. Note that endogenous p73 levels are unchanged following ΔNp63 inhibition. **E.** ΔNp63 mediates apoptosis by p73 dependent manner. Quantitation of annexin V- and/or PI-positive Abrams cells treated as in D, Error bars show standard deviation for two independent experiments. **F.** ΔNp63 binds p73. Abrams and OSA8 cells were transfected with HA- tagged p73 plasmid. After 48 hr of transfection, immunoprecipitation was performed by using HA- tag or IgG control antibodies. Following immunoprecipitation, extracts were analyzed by western blot with p63 antibody as shown. **G.** Both induction of Noxa and Puma are prerequisite for inducing apoptosis following inhibition of ΔNp63. To silence ΔNp63 expression, OSA16 cells were treated with 200 μg/ml Doxycycline in presence of indicated siRNAs for 72h and cell extracts were analyzed by western blot with antibodies as shown.

siRNA mediated downregulation of ΔNp63 induced expression of the proapoptotic proteins Puma and Noxa in canine OSA cells (Figure 3A and Supplementary Figure S3A). In normal epithelial cells, ΔNp63 functions as a transcriptional repressor of cell cycle regulatory genes that are positively regulated by p53 [26]. It is therefore possible that ΔNp63 promotes survival through either direct or indirect repression of proapoptotic genes regulated by p53. To test this, p53 function was modulated in Abrams cells through transduction with a lentivirus encoding a C-terminal truncated p53 fragment (p53DD) that functions as a potent dominant negative regulator of wild-type p53 [27]. As shown in supplementary Figure S3B, cells expressing p53DD did not upregulate p21 protein in the presence of DNA damage, indicating a loss of functional p53 activity. OSA cell lines expressing the p53DD fragment were then transfected with ΔNp63-directed or control siRNAs. No effect of p53DD on the ΔNp63-dependent induction of Puma or Noxa was observed (Figure 3B). Furthermore, p53DD expression did not alter the induction of cell death following ΔNp63 knockdown, as assessed by annexin V/PI staining (Figure 3C). These data indicate that p53 does not contribute to the apoptotic program initiated following loss of ΔNp63.

ΔNp63 has been also implicated as a repressor of p73-dependent transcription [28, 29]. Interestingly, both p53 and p73 have been identified as regulators of Puma and Noxa transcription [30–33]. Consequently, we asked whether ΔNp63 might promote survival through inhibition of p73 function. To test this hypothesis, Abrams cells were first infected with lentivirus constructs expressing control or shRNA directed against the p73 N-terminal transactivation (TA) domain. After selection of infected cells, down regulation of p73 expression was verified by Western blotting (Figure 3D). Targeting p73 by specific shRNA in the Abrams cells did not block p63 expression, as demonstrated in Figure 3D. As expected, ΔNp63 knockdown induced proapoptotic Puma and Noxa proteins in control cells (Figure 3D). In contrast, concurrent inhibition of ΔNp63 and p73 abrogated Puma and Noxa induction (Figure 3D). Furthermore, in contrast to control cells, inhibition of ΔNp63 in sh-p73 Abrams cells caused minimal cell death as assessed by annexin V/PI staining suggesting ΔNp63 mediates apoptosis by p73 dependent manner (Figure 3E).

There are several models that could explain the functional inhibition of p73 by ΔNp63. We first examined whether ΔNp63 knockdown increased expression of either the p73 mRNA or protein. No change was detected in either the p73 mRNA by qRT-PCR or p73 protein following ΔNp63 knockdown in both Abrams and OSA8 cells (Supplementary Figure S3C-S3D). The p63 and p73 proteins contain a highly homologous (>60% identical) oligomerization domain, and they are reported to interact physically when co-expressed [28, 34]. To assess the ability of p73 to interact with ΔNp63 in canine OSA, an HA tagged p73 plasmid was transfected into Abrams cells. As shown in co-immunoprecipitation experiments in Figure 3G, p73 and ΔNp63 are directly associated with one another suggesting that ΔNp63 suppresses apoptosis in canine OSA cell lines by directly binding to p73, thereby blocking p73-dependent suppression of pro-apoptotic proteins. Lastly, we asked whether Noxa and Puma mediate the induction of cell death. To test this hypothesis, we first generated doxycycline-inducible shΔNp63 cells. As shown in Figure 3G, induction of both Noxa and Puma were requisite for the induction of apoptosis following inhibition of ΔNp63. Similar results were obtained using secondary siRNA targeting of Noxa and Puma, as shown in Supplementary Figure S3E. This suggests that induction of Puma and Noxa by p73 is required for the apoptotic program initiated by loss of ΔNp63 in canine OSA cells.

### ΔNp63 drives angiogenesis by inducing IL8

**Figure 4:**
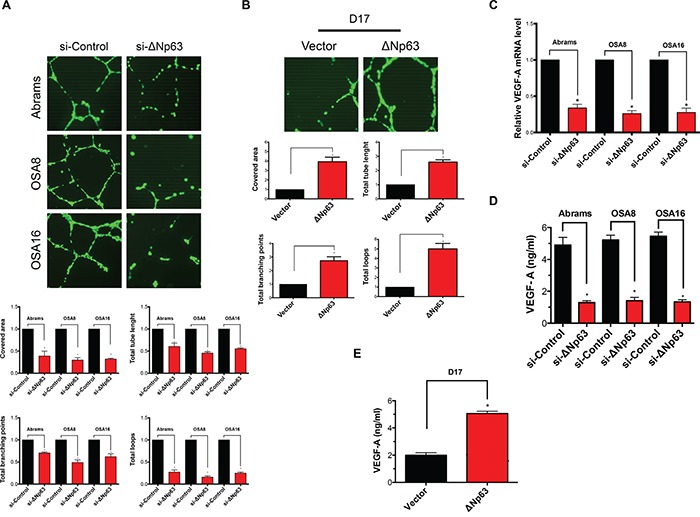
ΔNp63 induces angiogenesis **A.** Depletion of endogenous ΔNp63 in canine osteosarcoma cell lines results in a significant decrease of endothelial tube formation. Tube formation assay and the quantification of the tube formation ability of HUVEC cells were described in materials and methods. All values shown are expressed as the mean ± SD obtained from three independent experiments. *P < 0.05 versus control shRNA. Experimental data were normalized to the control. **B.** Introduction of ΔNp63 in D17 cells inducesendothelial tube formation. Tube formation assay and the quantification of the tube formation ability of HUVEC cells were described in materials and methods. All values shown are expressed as the mean ± SD obtained from three independent experiments. *P < 0.05 versus control vector. Experimental data were normalized to the control. **C.** qRT-PCR was used to assay VEGF-A mRNA levels. Results were first normalized to GAPDH and subsequently data are normalized to the control. Data shown are mean ± SD of triplicate measurements from one representative experiment. Data sets were analyzed with two-tailed student's t-test and significance between two groups is shown *P < 0.05. Experiment has been repeated 2 times with similar results. **D.** Inhibition of ΔNp63 in canine osteosarcomas reduces VEGF-A secretion. Cells were treated with either si-ΔNp63 or control siRNA for 48 hr. Subsequently, VEGF-A production in the media was determined as described in Materials and Methods. **E.** Overexpression of ΔNp63 in D17 cells inducesVEGF-A secretion. VEGF-A production in the media was determined as described in Materials and Methods.

ΔNp63 has been suggested to influence the process of angiogenesis. To determine whether modulation of ΔNp63 in canine OSA cells could impact angiogenesis in a transwell assay system, we used the human umbilical vein endothelial cell (HUVEC) tube forming assay. As shown in Figure 4A, depletion of the ΔNp63 isoform in canine OSA cell lines markedly reduced tube formation determined by quantification of covered area, total tubes, total tube length, total branching points and total number of loops. To further confirm these findings, D17 cells were stably infected with lentiviruses expressing either ΔNp63 or empty vector. After selection, stable expression of ΔNp63 in D17 cells was verified by Western blotting (Supplementary Figure S3F). In contrast to cells infected with empty lentiviral vector, stable overexpression of ΔNp63 in D17 cells stimulated HUVEC tube formation using the transwell assay system (Figure 4B).

Given that p63 isoforms are proteins with sequence-specific DNA-binding properties, we hypothesized that ΔNp63 might alter the expression of genes critical for angiogenesis. Next, we evaluated both gene expression level and the secreted protein level of vascular endothelial growth factor (VEGF-A) in canine OSA cells in the presence or absence of ΔNp63. Downregulation of ΔNp63 markedly reduced both VEGF-A expression (Figure 4C) and VEGF-A secretion by 48 hrs post siRNA transfection (Figure 4D). As significant apoptosis did not occur until 72 hours post transfection, our data indicate that the loss of VEGF-A was likely not a result of cell death. Furthermore, D17 cells transfected with ΔNp63 demonstrated significantly increased VEGF-A secretion in contrast to control D17 cells (Figure 4E). Taken together, our data suggest that ΔNp63 can modulate both VEGF-A expression and production in canine OSA cells potentially contributing to the observed effects of ΔNp63 on angiogenesis.

**Figure 5:**
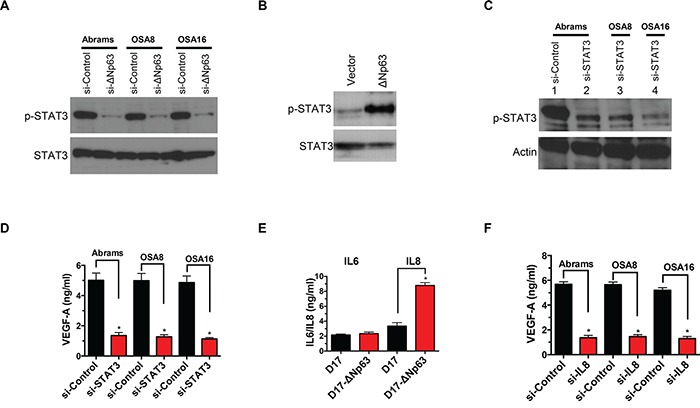
ΔNp63 activates VEGF-A secretion by STAT3 and IL-8 axis **A.** Inhibition of endogenous ΔNp63 abrogates STAT3 phosphorylation in canine osteosarcoma cell lines. After control or si-ΔNp63 transfection, *cells* were cultured in media containing *1%* FBS for *48 hr and* cell extracts were analyzed by western blot with antibodies as shown. **B.** Stable expression of ΔNp63 in D17 cells significantly increases STAT3 phosphorylation. *Cells* were cultured in media containing *1%* FBS for 48 hr *and* cell extracts were analyzed by western blot with antibodies as shown. **C.** Inhibition of STAT3 by specific siRNA was verified by western blot analysis. **D.** Inhibition of STAT3 in canine osteosarcoma correlates with decreased VEGF-A secretion. VEGF-A production in the media was determined as described in Materials and Methods. **F.** Stable expression of ΔNp63 in D17 cells significantly increases IL-8 secretion. IL-6 and IL-8 ELISA Kits were purchased from R&D Systems and performed according to the manufacturer's instructions. **E.** IL-8 mediates VEGF-A secretion in canine osteosarcoma cells. Cells were treated either si-ΔNp63 or the control siRNA for 48 hr. Subsequently, VEGF-A production in the media was determined as described in Materials and Methods.

Recent work in human OSA cells demonstrated that ΔNp63 stimulates both IL-6 and IL-8 secretion, leading to increased STAT3 phosphorylation (pSTAT3) and VEGF-A secretion through stabilization of HIF-1α [21]. Consistent with these findings, inhibition of ΔNp63 in canine OSA cell lines reduced pSTAT3 (Figure 5A), while overexpression of ΔNp63 induced pSTAT3 activity in D17 cells (Figure 5B). These findings support the notion that signaling pathway alterations fundamental to VEGF-A regulation are conserved between human and canine OSA at a molecular level. In support of this, siRNA mediated downregulation of total STAT3 in canine OSA cells was associated with a significant decrease in VEGF-A production (Figure 5C–5D). In contrast to human OSA, ΔNp63 induced IL-8 but not IL-6 secretion (Figure 5E and Supplementary Figure S4A). Furthermore, reduction of IL-8 secretion correlated with inhibition of ΔNp63 expression (Supplementary Figure S4B). Lastly, inhibition of IL-8 in canine OSA cells significantly decreased VEGF-A secretion (Figure 5F). Recent work has demonstrated that ΔNp63, RelA, and cRel members of the NF-κB family can interact to affect transcription of NF-κB/Rel target genes, including IL-8 and IL-6 [21, 35]. As shown in supplementary Figure S4C-S4D, abrogation of NF-κB family proteins significantly reduced IL-8 secretion, mimicking inhibition of ΔNp63 in OSA cells. This supports the notion that ΔNp63, in coordination with NF-κB family members, regulates IL-8 in canine OSA. Interestingly, in contrast to the IL-8 promoter, the IL-6 promoter in dogs does not contain an NF-κB binding site (Supplementary Figure S4E), suggesting that NF-κB/Rel regulatory elements are essential for the induction of IL-8 by ΔNp63. In summary, ablation of ΔNp63 in OSA cell lines markedly alters the pre-existing angiogenic phenotype through modulation of VEGF-A, IL-8 and pSTAT3. In contrast to human OSA, ΔNp63 indirectly drives STAT3 phosphorylation in an autocrine loop, by transactivating the cytokine IL-8, but not IL-6, which are upstream regulators of the c-Jun N-terminal kinase (JNK)–STAT pathway.

### ΔNp63 expression promotes invasion and lung metastases in canine osteosarcoma

**Figure 6:**
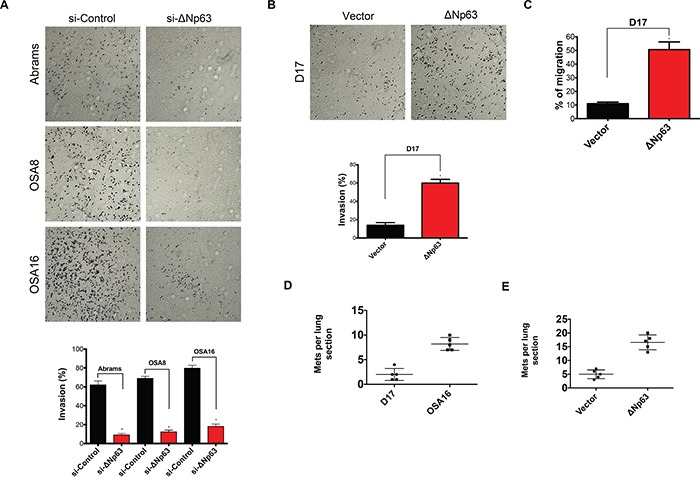
Expression of ΔNp63 plays a crucial role in invasion, cellular motility and progression of lung metastasis in canine osteosarcoma **A.** Inhibition of ΔNp63 reduces cellular invasion. Cells were treated with either si-ΔNp63 or control siRNA for 48 hr. Invasion assays were carried out using Matrigel precoated inserts (Corning). Representative fields were photographed. Assays were performed in triplicate for each treatment group and the results are expressed as migrated cells per field (below). Experiment is repeated one more time with similar results. **B.** Expression of ΔNp63 in D17 significantly increases cellular invasion. Representative fields were photographed as shown. Assays were performed in triplicate for each treatment group and the results are expressed as migrated cells per field (below). Experiment is repeated one more time with similar results. **C.** Expression of ΔNp63 in D17 significantly increases number of migratory cells. Quantitative migration assays were performed as described in material and methods section. Briefly, D17 cells were seeded in the top of the insert in serum-free media, while 0.1% FBS was used as a chemoattractant in the well below. Experiment is repeated one more time with similar results. **D.** Expression of ΔNp63 in canine osteosarcoma correlates with increased lung colonization. OSA16 is highly metastatic to the lung when inoculated via tail vein, whereas D17 cells form lung metastases with much lower frequency. 10^6^ cells from either D17 or OSA16 were injected into the tail veins of SCID mice (5/group). After 42 days, lungs were harvested, insufflated, fixed, sectioned, and stained. The numbers of lung sections with metastatic nodules were compared with the Mann–Whitney Utest. OSA16 cells generated three fold more metastases (*P* values <0.05). **E.** Stable overexpression of ΔNp63 in a poorly metastatic D17 cells increases lung colonization. 10^6^ cells from either D17-control vector or D17-ΔNp63 were injected into the tail veins of SCID mice (5/group). After 42 days, lungs were harvested, insufflated, fixed, sectioned, and stained. The numbers of lung sections with metastatic nodules were compared with the Mann–Whitney Utest. Introduction of ΔNp63 in D17 cells resulted in more than threefold metastases (*P* values <0.05).

Our data demonstrate that ΔNp63 promotes tumorigenesis in canine OSA by preventing apoptosis and inducing VEGF-A expression. To begin investigating the potential role of ΔNp63 in metastasis, we determined its influence on invasion and cell movement through extracellular matrices using a transwell invasion assay. As shown in Figure 6A, 48 hrs following downregulation of ΔNp63, the number of cells invading across the membrane were markedly reduced. In contrast, overexpression of ΔNp63 in D17 cells increased the number of cells invading across the membrane (Figure 6B). Furthermore, overexpression of ΔNp63 in D17 cells increased cellular migration (Figure 6C) and wound healing (Supplementary Figure S5A-S5B). Together, these data indicate that ΔNp63 may contribute to the metastatic phenotype in canine OSA by modulating cellular invasion.

To more directly evaluate the effects of ΔNp63 on metastasis, an *in vivo* approach was used. OSA16 and D17 cells expressing high or low levels of ΔNp63 were inoculated via tail vein injection into SCID mice. As shown in Figure 6D, high expression of ΔNp63 correlated with increased numbers of metastatic pulmonary nodules. To further strengthen this correlation, ΔNp63 overexpressing D17 and control D17 cells were subsequently inoculated via tail vein injection into SCID mice. As shown in Figure 6E, stable overexpression of ΔNp63 in D17 cells increased lung colonization. In summary, these data highlight the contribution of ΔNp63 to the process of metastasis.

## DISCUSSION

The expression and functional consequences of aberrant ΔNp63 expression were evaluated in canine OSA, an important spontaneous preclinical model of the human disease. Dysregulation of candidate tumor suppressor genes, such as *p53* and *PTEN*, as well as the oncogenes *MYC* and *MET* is found in both canine and human OSA [36, 37]. Furthermore, gene expression analysis has demonstrated the similarity between orthologous genes in people and dogs, supporting the notion that OSA in dogs and people is a genetically indistinguishable [9, 37]. There are many features critical for the outgrowth of metastatic tumor cells, and mounting evidence supports a role of ΔNp63 in the malignant phenotype of OSA in people [21, 38]. Therefore the study of ΔNp63 in canine OSA may lend insight into the complex biology of this disease in people.

It has been shown that p63 is essential for normal epithelial development in both mice and humans. Here, we demonstrate that ΔNp63 is significantly overexpressed in both primary canine OSA tumor samples and several canine OSA cell lines, and that ΔNp63 expression promotes the survival of OSA tumor cells by virtue of its ability to suppress p73-dependent apoptosis, induce invasion, cellular motility, and progression of pulmonary metastatic disease. Further investigation is warranted to delineate this process, however inherent differences in the clinical course of canine and human OSA lend insight into the role of ΔNp63. While OSA is the most common primary bone tumor in both people and dogs, the prevalence of this disease is strikingly higher in dogs compared to people, with a reported incidence of 13.9/100,000 in dogs and 1.02/100,000 in people [4, 39]. Despite amputation and adjuvant chemotherapy, the clinical course of canine OSA is more aggressive, with 90% of dogs euthanized within 2-years of diagnosis. In contrast, 60-70% overall 5-year survival rates are reported in non-metastatic human OSA.

Consistent with the observation that canine OSA tends to exhibit a more aggressive clinical course than its human counterpart, we found that over-expression of ΔNp63 occurred in substantially more primary canine tumor specimens (nearly all tested) compared to that described in human primary tumors [21]. Furthermore, the canine tumor cell lines exhibiting high ΔNp63 expression demonstrated a resistance to apoptosis and generated significantly more metastatic lesions *in vivo*. These data suggest that ΔNp63 overexpression likely contributes to the observed aggressive behavior of OSA in dogs, including the rapid development of chemotherapy resistant metastases. The difference in ΔNp63 expression patterns between humans and dog OSA provides a unique opportunity to interrogate the mechanisms that regulate ΔNp63 in the canine disease. For example, it is possible that polymorphisms within the internal promoter region of p63 contribute to high expression of ΔNp63 in canine OSA, thereby promoting a more aggressive biologic behavior. In support of this possibility, it was recently demonstrated that interindividual differences in internal promoter region haplotypes influence baseline Δ133p53 expression, and that polymorphisms within the internal promoter of the p53 gene drive the overexpression of Δ133p53, an oncogenic isoform of that gene [40]. Interestingly, STAT3 is constitutively phosphorylated in many cancers including canine and human OSA [41]. For example, up-regulation of ΔNp63 was correlated with the activation of STAT3 in human nasopharyngeal carcinoma cells [42]. The blockade of STAT3 nuclear translocation abolished its up-regulation by ΔNp63, suggesting that a positive feedback loop between ΔNp63 and STAT3 activation may be present in canine OSA.

There are many cell stressors that can trigger the apoptotic response pathway, including genome instability, oncogenic stress, and hypoxia, among others [43, 44]. Inactivation of pathways mediating apoptosis is an essential hallmark of tumor cells [45]. One common mechanism for disabling apoptosis involves inactivation of the tumor suppressor gene p53 [46, 47]. Interestingly, our results show that inhibition of ΔNp63 results in p53-independent, but p73-dependent apoptosis, indicating that p53 is not a driver of apoptosis in the canine OSA cell lines evaluated. While the underlying mechanism resulting in these findings was not apparent, considerations that warrant further investigation include evaluation of canine OSA cell lines for mutations in p53, or inactivation of p53 as a result of MDM2 overexpression. The coding regions of *p53* are highly conserved between dogs and humans [48] and the reported frequency of p53 mutations is similar in canine and human OSA, ranging from 23-47% in dogs and 15-30% in people [36, 49–51].

In contrast to p53, the high sequence homology within both the DNA binding and oligomerization domains of ΔNp63 and p73 could endow ΔNp63 with the ability to significantly inhibit p73 activity. In support of this, we found that p73 is complexed to endogenous ΔNp63 in OSA cells. Of note, ΔNp63 contains a DNA-binding domain and recognizes a similar set of promoter regions compared to p53, including those for Puma and Noxa, both crucial pro-apoptotic members of the Bcl-2 family [30, 31]. It is therefore possible that ΔNp63 can directly interact with Puma or Noxa promoters to inhibit p73-dependent transcription.

As previously indicated, inactivation of p73 by ΔNp63 may be an essential determinant of the malignant phenotype in OSA. However, cancer involves complex genetic and environmental stimuli, and resisting apoptosis represents only one of the fundamental roadblocks to tumorigenesis [45]. As tumor cells multiply, the tumor microenvironment becomes progressively hypoxic. A distinctive feature of neoplastic cells is their ability to flourish in a variety of tumor microenvironments through acquisition of multiple hallmarks of malignancy, including dysregulation of their cellular energetics and the induction of angiogenesis, among others. For example, copy number gains at CFA13 in canine OSA, including the *MYC* oncogene were among the most frequent copy number aberrations identified in one study. Myc activates target genes involved with cell growth and proliferation, and in some tumor cells Myc can upregulate expression of angiogenic factors. Taken together, this underscores the concept that oncogenes driving cell proliferation can also induce angiogenesis [45].

Our data demonstrate that ΔNp63 drives tumors to express VEGF-A. Interestingly, we also showed that increased VEGF-A production in canine OSA is linked to IL-8, but not IL-6 dependent mechanisms. Previously published data elucidated the interaction of NF-κB family members (RelA and cRel) and ΔNp63, resulting in transcription of NF-κB/Rel target genes in human cells. Although this model needs to be further evaluated in canine cells, our data indicate that the IL-6 promoter region in canine OSA does not contain an NF-κB binding motif. Interestingly, in contrast to IL-8, we detected very low IL-6 secretion, suggesting that IL-8 is a major driver of angiogenesis in canine OSA. Alternatively, other cytokines known to drive gp130 mediated signal transduction resulting in STAT3 phosphorylation and VEGF-A production could be involved. Previous work has demonstrated that canine OSA cell lines express gp130, but not the IL-6R and they do not respond to IL-6. However, they do express the Oncostatin M (OSM) receptor, produce OSM and respond to exogenous OSM through enhanced STAT3 phosphorylation [41]. Furthermore, IL-11Ra is also expressed on canine OSA cells, so IL-6 cytokine family members such as IL-11 and OSM may be drivers of ΔNp63-mediated interactions in the canine disease.

The ability to promote angiogenesis is thought to be one of the primary mediators of metastasis in OSA. Ultimately, metastatic disease is responsible for the majority of patient deaths as it remains refractory to standard and experimental approaches to therapy. We found that up-regulation of ΔNp63 correlates with invasion, cellular motility and progression of lung metastasis in canine OSA tumor cell lines and in mouse models of disease. It is possible that ΔNp63-mediated IL-8 upregulation is responsible for the observed increase in pulmonary metastasis. Alternatively, other cytokines previously mentioned (OSM, IL-11) may also play a role. Of note, the basic steps of metastasis consist of local invasion, intravasation, survival in the circulation, extravasation and colonization [52]. The identity and time of onset of the changes that endow tumor cells with these metastatic capabilities are largely unknown and are still subject to debate [52]. Our data show that ΔNp63 expression may be an important component of this process. However, further efforts directed at elucidating the underlying molecular mechanisms by which ΔNp63 expression influences the metastatic cascade in OSA are indicated. In summary, our data emphasize the importance of ΔNp63 in the invasion, cellular motility and progression of pulmonary metastasis, and provide support for the investigation of ΔNp63 as a potential target for therapeutic intervention in human and canine OSA.

## MATERIALS AND METHODS

### Animal studies

All animal experiments were conducted in accordance with institutional animal care and use committee of the research Institute at Nationwide Children's Hospital. Approved protocols were designed to minimize the numbers of mice used and to minimize any pain or distress. 10^6^ cells were suspended in 100 μl of 1xPBS and injected into the tail veins of 6-8 week-old CB17SC scid^−/−^ female mice (Taconic Farms). After six weeks, lungs were harvested, insufflated, fixed, sectioned, and stained. Number of metastases per section was quantified by light microscopy. All mice were maintained under barrier conditions.

### Cell culture

The Abrams cell line was maintained in DMEM supplemented with 10% FBS, non-essential amino acids, sodium pyruvate, penicillin, streptomycin and L-glutamine (Corning). OSA8, OSA16 and D17 cell lines were maintained in RPMI supplemented with 10% FBS, 0.1 mM non-essential amino acids, sodium pyruvate, penicillin, streptomycin, L-glutamine (Corning). Cells were maintained in monolayer culture at 37°C in humidified air with 5% CO2 in these growth media.

### Western blot and immunoprecipitation

Cells were resuspended in 1xRIPA lysis buffer (Cell Signaling Technology) supplemented with protease and phosphatase inhibitors (Thermoscientific), and 1 mM PMSF (Sigma). Subsequently, sonication (Misonix) is used for lysing resuspended cells on ice. After protein concentrations were determined using Biorad protein assay and measuring absorbance at 595 nm on a Backman Coulter, DU 800 Series UV/Vis Spectrophotometer, proteins were separated by using Novex™ NuPAGE^®^ Gel Electrophoresis Systems (Invitrogen), transferred to nitrocellulose membranes by iBlot^®^ Gel Transfer Device (Invitrogen) and incubated with following primary antibodies: β-Actin, p53 and p63 (4A4) (Santa Cruz), STAT3, pSTAT3 (Tyr-705) and PARP (Cell Signaling Technology), p73 (NeoMarkers, cocktail *Ab*-4), PUMA and IkB-alpha (Abcam) and Noxa (ProSci incorporated). Immunoprecipitation was performed with Dynabeads^®^ (Invitrogen). Cell lysates were incubated with Anti-HA-Tag rabbit polyclonal antibody (Sigma-Aldrich) for 24h. Subsequently, lysates were incubated with pre-cleared Dynabeads^®^ for 4h. After washing steps, western blot was performed with anti-p63 (4A4) antibody (Santa Cruz).

### RNA isolation, cDNA synthesis, and real time PCR (RT-PCR)

RNA isolation and reverse transcription were performed using the RNeasy Mini Kit and Omniscript Reverse Transcriptase according to the manufacturer's instructions (Qiagen). qPCR was performed on an ABI Prism 7900HD Sequence Detection System (Applied Biosystems) using the TaqMan Universal Mastermix (Applied Biosystems). VEGF-A TaqMan^®^ Gene Expression Assays was purchased from Applied Biosystems. ΔNp63 expression was quantified in real-time with a specific FAM-labeled MGB-probe (Applied Biosystems, Forward Primer: GAGGGACTTGAGTTCTGTTATCT, Probe: AGATTCCTATTGTCAGGGTCTCAGAGGG, Reverse Primer: CTCTTCTGGCTCCAGGATTT) and normalized to GAPDH (Applied Biosystems, Forward Primer: GATGGGCGTGAACCATGAG, Probe: CCCTCAAGAT TGTCAGCAATGCCTCCT, Reverse Primer: TCATG AGGCCCTCCACGAT).

### Plasmids, lentiviral production and siRNAs

ΔNp63 sequence subcloned from pcDNA3.1-ΔNp63 (Addgene, Plasmid #26979) into the pLenti-CMV vector. For inducible ΔNp63 knockdown, specific ΔNp63 shRNA sequence (GGAAAACAATGCCCAGACT) cloned into pLKO-Tet-Neo plasmid (Addgene). p53DD and control lentiviral plasmids were described in [53]. The lentiviral constructs and high-titer lentiviral stocks were generated as described in Addgene's pLKO.1 protocol. The STAT3 siRNA was purchased from Cell Signaling Technology and all other of siRNAs oligos used in this study were purchased from Dharmacon and transfected with Lipofectamin 2000 (Invitrogen) into osteosarcoma cell lines. The target sequences of short hairpin RNAs (shRNAs) for p73 and all RNAi oligos used in this study are listed in Supplementary Table S1.

### Invasion and quantitative migration assay

Invasion assays were carried out using matrigel invasion chamber following the manufacturer's instructions (Corning). 600 μl of media with 2% FBS was placed in the lower wells. 100 μl of cell suspension (2×10^5^ cells/ml) was loaded into each of the upper wells with medium containing 0.1% FBS. The chambers were incubated for 24 hr at 37°C. After incubation, the inserts were removed, and the non-invading cells on the upper surface were removed with a cotton swab. The cells on the lower surface of the membrane were fixed and stained with Diff-Quik stain kit (Siemens) following manufacturer's instructions. Subsequently, invaded cells were imaged under the microscope at 40X magnification and counted in several fields in triplicate. Finally, the percent invasion was determined as described in the Corning's protocol (Cell Invasion Assay). Each experiment was performed at least two times. For the quantitative migration assay, corning control inserts (contains no Matrigel) were used. 100 μL cell suspensions containing 0.1% FBS were seeded in the upper chamber. To attract the cells, 600 μL of Medium with 2% serum was added to the bottom chamber. After allowing the cells to migrate for 24 h, the penetrated cells on the filters were fixed by using Diff-Quik stain kit (Siemens). Migration was quantified using the ratio of the migrated cells over the total cells (migrated plus remaining cells) to determine the fraction of migrated cells in each individual experiment. Each experiment was performed at least two times.

### Transwell endothelial tube formation assay

The ECM gel (Cell Biolabs) was thawed on ice and mixed to homogeneity using cooled pipette tips. Cell culture plates (24-well) were bottom-coated with a thin layer of ECM gel (280 μl/well), which was left to polymerize at 37°C for 60 min. HUVEC cells (4×10^4^ cells) were added to each well on the solidified ECM gel. 100 μl of cell suspension (2×10^5^ cells/ml) was loaded into each of transwell filter inserts (pore size 0.4 μm; Corning) with medium containing 0.1% FBS. The chambers were incubated for 24 hr at 37°C. After incubation, the inserts were discarded, and HUVEC cells were stained with Calcein AM (Cell Biolabs). Three microscope fields per treatment were photographed and, images were processed by using WimTube software (Wimasis GmbH). Subsequently, the tube formation ability of HUVEC cells were determined by quantification of covered area, total tubes, total tube length, total branching points and total number of loops by using WimTube software (Wimasis GmbH). Results were normalized to the controls.

### Wound healing assay

Cell migratory ability was measured using the scratch assay. Briefly, cells were seeded into six-well plates in medium containing 10% FBS and cultured to form a confluent monolayer. Linear wounds were created in the cell monolayers using a sterile pipette tip. Images were captured and documented at 0 and 32 h after wounding using a microscope (AMG, EVOS xl core) with a 4× objective lens. To determine the scratch area, three images per treatment were analyzed by using WimScratch software (Wimasis GmbH).

### Quantitative ELISA and annexin V-FITC staining

VEGF-A, IL-6 and IL-8 were detected by using canine Quantikine ELISA Kit according to the manufacturer's instructions (R&D Systems). For the detection of apoptosis, cells were first transfected at 50% confluence with control or siRNA targeting ΔNp63 and incubated for 72h. Subsequently, cells were washed with PBS and stained with FITC-Annexin V and PI according to the manufacturer's instructions (Leinco Annexin V Kit) and analyzed by flow cytometry.

### Statistical analysis

Error bars represent mean ± SD from triplicate measurements from one experiment. One representative experiment is showing. Experiments have been repeated at least two times with similar results. Differences between two groups were analyzed by Student's *t*-test. The numbers of lung sections with metastatic nodules were compared with the Mann–Whitney U test. P values of ≤ 0.05 were considered statistically significant.

## SUPPLEMENTARY FIGURES AND TABLE


