# Supplementary material for: ΔNp63 mediates cellular survival and metastasis in canine osteosarcoma
Source: Oncotarget. 2016 Jul 6;7(30):48533–46. doi: 10.18632/oncotarget.10406 (PMC5217036; doi:10.18632/oncotarget.10406)
Supplement: Supplementary file 1 [file oncotarget-07-48533-s001.pdf]

ΔNp63 mediates cellular survival and metastasis in canine osteosarcoma

SUPPLEMENTARY FIGURES AND TABLE

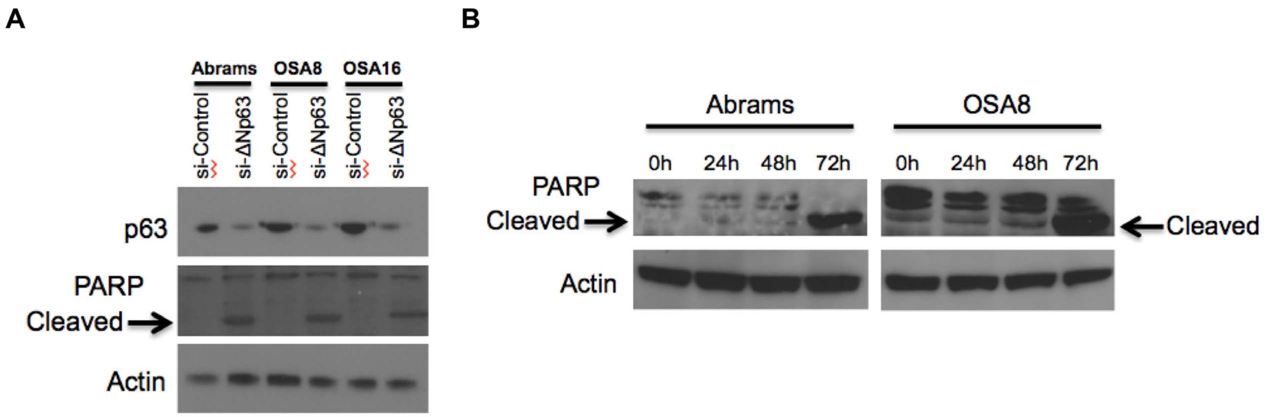

Supplementary Figure S1: A. Silencing of ΔNp63 in canine OSA cell lines induces apoptosis. Induction of apoptosis in cell lines was determined by the presence of cleaved PARP an indicator of apoptosis. Cell extracts were harvested from indicated cell lines 72 hr following infection with si-ΔNp63 or control siRNA and analyzed by western blot with antibodies as shown. **B.** Inhibition of ΔNp63 has no significant effects on apoptosis at early time points. The absence of cleaved PARP in 24–48 hr confirms that inhibition of ΔNp63 results in apoptosis at late stage. As shown above, knockdown of ΔNp63 induces PARP cleavage in both Abrams and OSA8 cells at 72 hr. Cell extracts were harvested from indicated time points following infection with an si-ΔNp63 or control siRNA and analyzed by western blot with antibodies as shown.

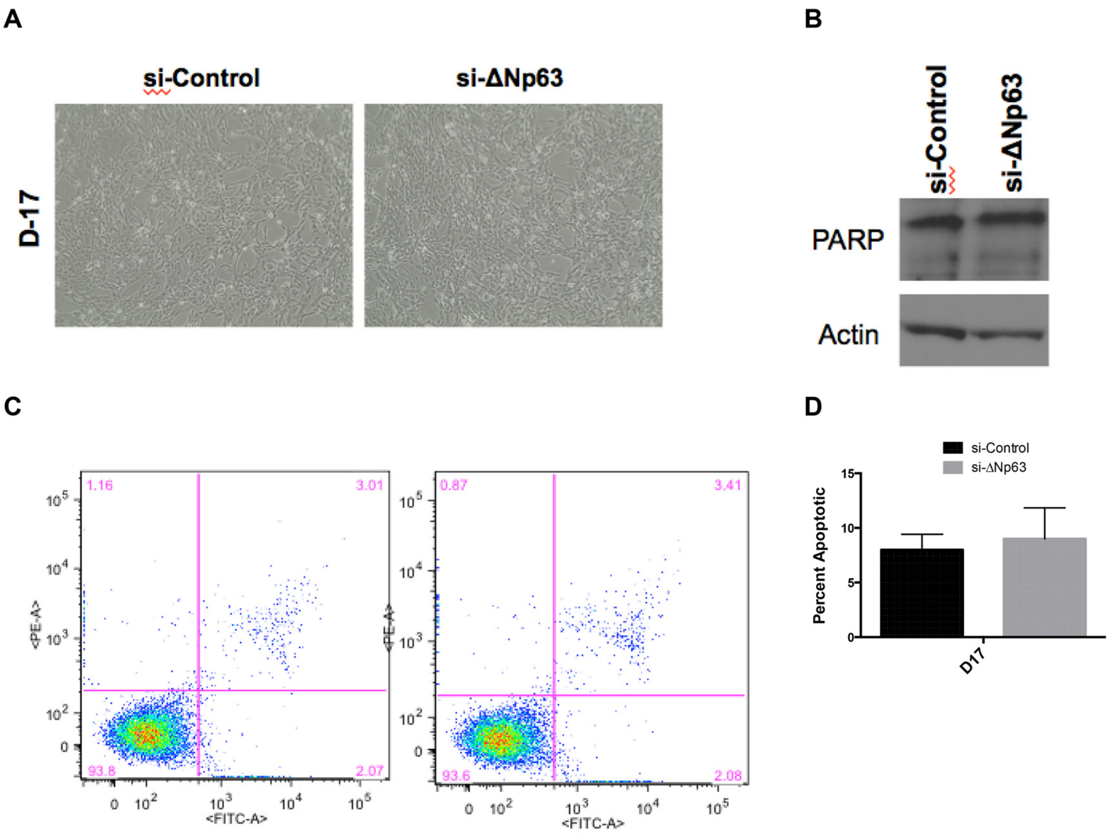

Supplementary Figure S2: Inhibition of ΔNp63 in D17 cells has no effects on apoptosis. A. A photographed representative field showing no cell death 72 hr following si-ΔNp63 transfection in D17 cells. **B.** Knockdown of ΔNp63 did not induce PARP cleavage in D17 canine osteosarcoma cells. Cell extracts were harvested from D17 cell lines 72 hr following infection with si-ΔNp63 or the control siRNA and analyzed by western blot with antibodies as shown. **C.** No significant induction of apoptosis in D17 canine osteosarcoma cells following infection with either si-ΔNp63 or the control siRNA. Unfixed cells were stained with annexin V and propidium iodide (PI) 72 hr following transfection with the indicated siRNAs, then analyzed by flow cytometry. Numbers refer to the percent annexin V- and/or PI-positive cells (UL + UR + LR quadrants) in this representative experiment. **D.** Quantitation of annexin V- and/or PI-positive cells treated and analyzed as in C. Data shown are mean ± SD of triplicate measurements from one representative experiment. Data sets were analyzed with two-tailed student’s t-test and no significance between two groups found. Experiment repeated one more time with similar result.

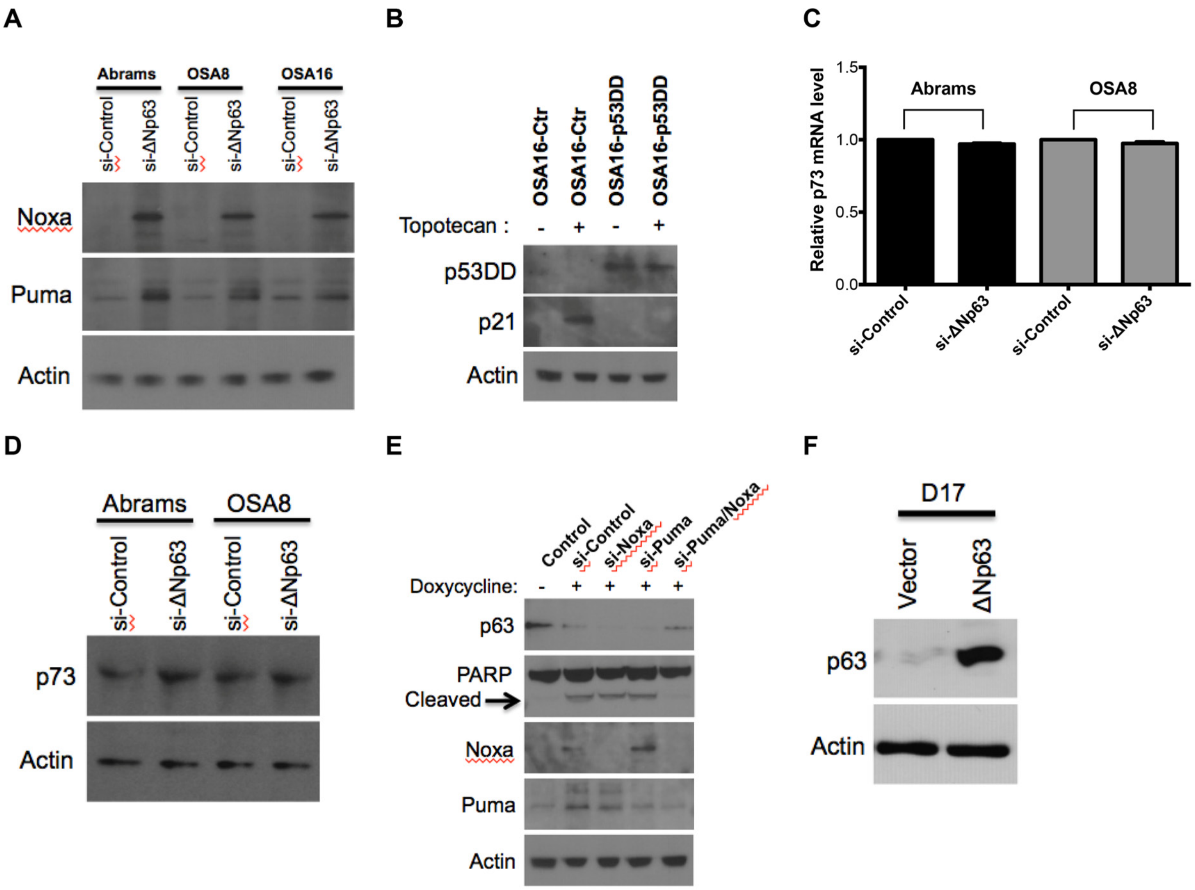

Supplementary Figure S3: Silencing of ΔNp63 in canine OSA cell lines induces both Puma and Noxa proteins. A. Cell extracts were harvested from indicated cell lines 72 hr following infection with si-ΔNp63 or the control siRNA and analyzed by western blot with antibodies as shown. **B.** Inhibition of p53 in OSA16 by dominant negative protein abrogates p21 protein induction following topotecan treatment. **C-D.** Both transcriptional and translational levels of p73 are not affected following inhibition of ΔNp63. C. qRT-PCR was used to assay p73 mRNA levels in indicated canine osteosarcoma cell lines relative to control siRNA treated cells. Total RNA was reverse-transcribed and subjected to real-time PCR with probes specific for p73. Results were first normalized to GAPDH and subsequently data are normalized to the control. Data shown are mean ± SD of triplicate measurements from one representative experiment. Data sets were analyzed with two-tailed student's t-test and no significance between two groups found. D. No significant changes at p73 protein level were observed following infection with either si-ΔNp63 or the control siRNA. Cell extracts were harvested from indicated cells following infection with si-ΔNp63 or control siRNA and analyzed by western blot with antibodies as shown. **E.** Both induction of Noxa and Puma are prerequisite for inducing apoptosis following inhibition of ΔNp63. To silence ΔNp63 expression, OSA16 cells were treated with 200 µg/ml Doxycycline in presence of indicated siRNAs for 72h and cell extracts were analyzed by western blot with antibodies as shown. **F.** D17 cell were infected with lentiviruses expressing either ΔNp63 or the control pLenti-CMV vector. After selection, expression of ΔNp63 was verified by western blot with p63 antibody as shown.

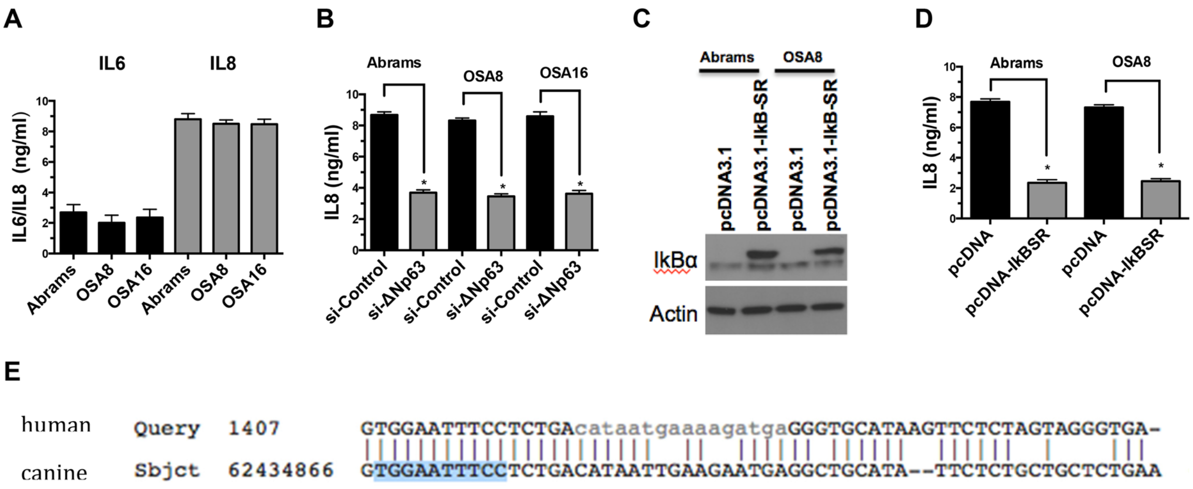

Supplementary Figure S4: ΔNp63 induces IL-8 secretion. A. Comparison of IL-6 and IL-8 secretion in canine osteosarcoma cells. IL-6 and IL-8 ELISA Kits were purchased from R&D Systems and performed according to the manufacturer's instructions. **B.** IL-8 secretion is significantly abrogated following ΔNp63 inhibition. IL-8 production in the media was determined according to the manufacturer's instructions (R&D Systems). **C.** Abrams and OSA8 cells were transfected with indicated plasmids. The expression of IκBα-*super repressor* (SR) was determined by western analysis. **D.** Inhibition of NF-κB by expression of SR reduced significantly IL-8 secretion. IL-8 ELISA Kit was purchased from R&D Systems and performed according to the manufacturer's instructions. **E.** Alignment of human and canine IL-8 promoters. NF-κB binding motive (TGGAATTTCC) is present in both human and canine IL-8 promoters.

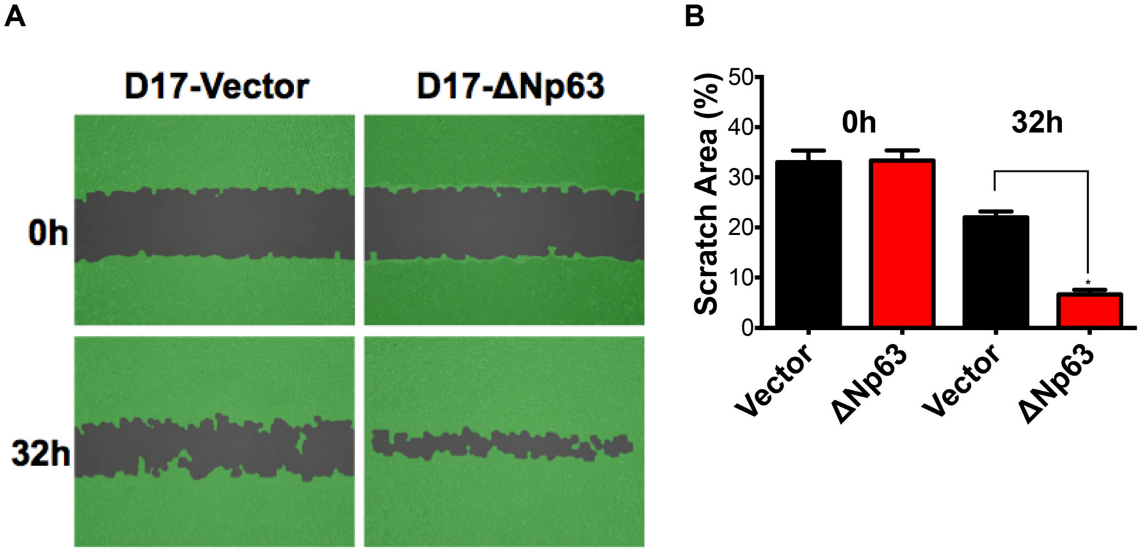

Supplementary Figure S5: Overexpression of ΔNp63 in D17 cells improved wound-healing. A. Wound-healing assay was used to evaluate the migration of D17 cells in the presence and absence of ΔNp63. Images are taken immediately after scratching the cultures 0h and 32h later and processed by WimScratch software (Wimasis GmbH). The image displayed is one of the representative results. **B.** To determine the scratch area, three images per treatment were analyzed by using WimScratch software (Wimasis GmbH). All values shown are expressed as the mean ± SD obtained from three independent experiments. *P < 0.05 versus empty vector in 32h.

Supplementary Table S1: Sequences for shRNA and RNAi oligos

Gene	Target sequence
p73 (shRNA)	GGCCATGCCCGTCTACAAG
ΔNp63 (siRNA 1)	GGAAAACAATGCCCAGACT
ΔNp63 (siRNA 2)	AAAACAATGCCCAGACTCA
Noxa (siRNA 1)	ATTTGGAGACAAACTGAAT
Noxa (siRNA 2)	GAATCTGTTATCCAAACTC
Puma (siRNA 1)	GGGTCCTGTACAATCTCAT
Puma (siRNA 2)	GGAGATGGAGCCCAATTAG
Control (siRNA)	ATCGCGTATAATACGCGTT
